# Vitamin D Binding Protein and Bone Health

**DOI:** 10.1155/2014/561214

**Published:** 2014-06-01

**Authors:** Ishir Bhan

**Affiliations:** Massachusetts General Hospital, Harvard Medical School, 5 Suite 750, 50 Staniford Street, Boston, MA 02114, USA

## Abstract

Vitamin D binding protein (DBP) is the major carrier protein of 25-hydroxyvitamin D (25(OH) D) in the circulation, where it may serve roles in maintaining stable levels during times of decreased 25(OH) availability and in regulating delivery of 25(OH) D to target tissues. Several genetic polymorphisms of DBP have been described that lead to phenotypic changes in the protein that may affect affinity, activity, and concentration. These polymorphisms have been linked with alterations in bone density in several populations. One of the mechanisms by which DBP may alter bone health involves regulating vitamin D bioavailability. DBP-bound vitamin is thought to be relatively unavailable to target tissues, and thus alterations in DBP levels or affinity could lead to changes in vitamin D bioactivity. As a result, functional vitamin D status may differ greatly between individuals with similar total 25(OH) D levels. Additionally, DBP may have independent roles on macrophage and osteoclast activation. This review will summarize recent findings about DBP with respect to measures of bone density and health.

## 1. Introduction

Vitamin D binding protein (DBP) is a 58 kDa circulating alpha globulin produced primarily by the liver. While initially known as Gc-globulin (group-specific component of serum), it has been renamed for its ability to bind the vast majority (>85%) of circulating 25-hydroxyvitamin D (25(OH) D). DBP is a member of the same protein family as albumin and is produced at relatively stable levels throughout life, though high estrogen states like pregnancy can promote increased production [1]. While it is best known for its vitamin D binding properties, it may have roles in other biological processes as well. Additional actions attributed to DBP include binding of extracellular actin and transport of fatty acids. DBP also appears to protect the complement factor C5a from proteolytic degradation, effectively enhancing its action as a chemotactic protein [2]. A deglycosylated form of DBP, DBP-macrophage activating factor (DBP-MAF), is able to promote activation of macrophages and osteoclasts, and even native DBP may have effects on osteoclasts [3]. Given both its vitamin D binding characteristics and its potential direct actions on bone resorption, considerable interest has been generated in the scientific community surrounding the potential actions of DBP on bone metabolism and health. Fueling this interest has been the discovery of significant interindividual differences in DBP levels. Early data points to associations between differences in DBP levels and bone density [4]. While some differences in DBP can be explained based on clinical characteristics, a moderate amount of variation in both levels and action appears to be driven by genetic polymorphisms.

## 2. Polymorphisms of DBP

Although over 120 variant forms of DBP have been recorded [5], three main phenotypic alleles have been described in the literature, initially identified based on their electrophoretic migration pattern. The slowest migrating is GC2, followed by GC1S (slow) and GC1F (fast) [6]. These phenotypic variants differ in both the associated concentration of DBP in the serum and their affinity for 25(OH) D and possibly other characteristics (see Table 1) [7]. In addition, there is substantial racial and geographic variation with these different forms. GC1F, which is associated with the lowest DBP concentration when present in homozygotes (though controversy remains about this topic), is more common in dark-skinned individuals, particularly those of African descent, while GC2 is more common in Caucasians [1]. The structural differences in these polymorphic forms are quite limited. Gc1S and GC2 differ from Gc1F only by single amino acid differences, tracked to single nucleotide polymorphisms. The rs7041 polymorphism leads to a substitution of g for t and, thus, a glutamate for an aspartate in G1S. The rs4588 polymorphism substitutes a for c and, thus, lysine for threonine in GC2 [8]. As each individual has two copies of the DBP gene, the combination of these various alleles may influence the levels and behavior of DBP on target tissues, including bone. Most studies of DBP's effects on bone in humans have focused on characterizing these allelic variants and attempting to correlate them with bone health.

## 3. DBP Polymorphisms and Bone Health

As women are at highest risk for the development of osteoporosis later in life, studies of DBP and bone disease have been concentrated in women. One of the first studies, originating over 20 years ago, looked at DBP in 258 nonblack elderly women, aged 65–90  [11]. The subjects were randomly selected as part of a prospective study of falls and fractures and underwent bone densitometry measurements at three sites (calcaneus, proximal radius, and distal radius) as well as height measurement. Phenotyping of DBP was performed based on electrophoretic analysis. Over two-thirds of the individuals in this study had either Gc1S/1S or Gc1S/2 diplotype, with less than three percent carrying the lowest-DBP allelic combination, Gc1F/1F. This lack of heterogeneity may have limited the ability of the authors to detect clinical differences between these alleles and, indeed, no statistically significant association with these allelic forms was found for bone mineral density (BMD), with respect to height or with respect to other bony measurements (os-calcis area and elbow breadth), even after multivariable adjustment for age and obesity.

Several more recent studies, however, are at odds with these early findings. While most studies have focused on the two major SNPs (rs7041 and rs4588) seen in Caucasians and people of African descent, Ezura et al. examined 13 DBP polymorphisms in a cohort of 384 postmenopausal Japanese women [12]. Five of these variants were significantly associated with BMD; one of these SNPs (−39*C* > *T*) was found in the promoter region, while the remainder were intronic. The combination of the exonic D432E (rs7041, which defines GC1S) with another SNP (IVS1 + 827*C* > *T*) was most strongly associated with BMD, suggesting that examining multiple SNPs simultaneously may be optimal for determining the effects of these polymorphisms on bone health, at least in some populations. Neither D432E nor T436K (rs4588) was significantly associated with DBP when examined in isolation in this population.

Studies in Western populations have yielded slightly different results. Lauridsen and colleagues used isoelectric focusing to determine Gc phenotype in 595 postmenopausal white Danish women as part of the Danish Osteoporosis Prevention Study, a 20-year partly randomized multicenter study of osteoporotic fracture prevention with hormone replacement therapy [13]. They identified large populations of GC1/GC1 and GC1/GC2 and a smaller population of GC2/GC2. They examined associations of these phenotypes with a historical evaluation of fracture number (prior to enrollment in the cohort), baseline bone mineral content (BMC), and baseline BMD at various sites (including forearm, lumbar spine, and hip). Subjects with GC1/GC1 were the most likely to have had a fracture, while GC2/GC2 subjects had the least, with GC1/2 individuals falling into an intermediate group. The effects were particularly pronounced for low-energy fractures. Despite these historical clinical associations, there neither were differences in measured BMC or BMD between the subgroups nor were there any differences in circulating markers of bone turnover. The authors also examined the effects of plasma DBP concentration; while no effect was seen in the overall population, DBP concentrations were negatively correlated with BMD at all sites in women who had a history of fractures. These results suggest that there may be subpopulations in which DBP phenotype is particularly important. Notably, a separate analysis of this cohort demonstrated unusually high DBP levels in subjects with GC1F/GC1F, conflicting with more recent data [10]. This discrepancy may reflect changes in DBP assays over time or other biological differences in the relatively small number of individuals with this phenotype (*n* = 17).

While the mechanism underlying the observed associations was not clear, a subsequent analysis found that levels of both 25(OH) D and 1,25-dihydroxyvitamin D (1,25(OH)_2_ D) were lower in GC2/GC2 individuals compared with GC1/GC1 (with GC1/GC2 again falling between the two) [14]. Another study of 741 European white women similarly examined the relationship between the two common DBP SNPs and 25(OH) D concentrations [15]. This study similarly found that the SNP variant associated with GC2 (rs4588) was associated with lower plasma 25(OH) D and also found similar results for the polymorphism at rs7041 associated with GC1F. However, DBP polymorphisms explained only 2% or less of the variation in 25(OH) D levels, similar to the amount explained by vitamin D intake. A third study extended these findings to infants and toddlers. Carpenter et al. studied the relationship between DBP polymorphisms and 25(OH) D levels in over 750 urban US children, 6–36 months of age [8]. Children were genotyped based on the two most commonly studied SNPs (rs7041 and rs4588). Only the rs4588 SNP appeared to be associated with 25(OH) D levels. When assessed by diplotype, genetic variation in DBP did appear to be associated overall with variation in 25(OH) D level, though the effect was again mild. As with prior studies, the polymorphism associated with the GC2 phenotype was linked with lower 25(OH) D levels. The authors also concluded based on multivariate analysis that only some of the genotypic associations of DBP with 25(OH) D could be attributed to differences in plasma DBP concentrations. Additional factors may be differences in affinity for 25(OH) D between the phenotypes or in other aspects of DBP action.

A recent study examined associations of DBP genotype with 25(OH) D levels, parathyroid hormone (PTH) levels, and bone density in a group of 231 Finnish children aged 7–19 [16]. Genotyping was done based only on rs4588, which identifies the GC2 phenotype as distinct from the GC1 forms, though only 6% of the study population was homozygous for GC2. GC2 homozygosity was again associated with the lowest 25(OH) D levels. DBP levels were not examined. The authors found that the effect of DBP genotype differed by gender. GC1 variants were associated with higher bone density only in boys, though another measure of bone health (strength and strain index) was significant in both genders. As expected, 25(OH) D levels were generally inversely associated with PTH levels, including within genotypes. Despite this, though GC2 homozygotes had the lowest 25(OH) D levels, they also had the lowest PTH levels. The authors hypothesize that this may reflect differential amounts of bound versus unbound 25(OH) D as a fraction of total 25(OH) D, thus affecting the PTH-vitamin D relationship. How this affects bone health itself is still unclear. Of note, rs4588 is located in exon 11, which is thought to affect the non-vitamin D binding activities of DBP, including potential effects on macrophage and osteoclast activation as part of DBP-MAF activity, so the effects on vitamin D biology may not be the only relevant factor to changes in bone density.

The gender differences in the association of DBP with BMD have also been seen in adult populations. Xu et al. studied 1873 individuals from 405 Caucasian European families, using the newer metric of compression strength index (CSI), which accounts for weight and the periosteal diameter of the femoral neck in the interpretation of femoral BMD [17]. Unlike most studies of DBP polymorphisms, the authors included 12 SNPs in the analysis, none of which were the common rs7041 and rs4588. Two were found to be significantly associated with CSI (rs222029 and rs222020; both are intronic), but gender-specific analysis revealed that these associations were driven entirely by men. The authors hypothesize that this may be due to a greater association between 25(OH) D and muscle strength in males, but the exact reason for the observed gender differences in this and other studies remains elusive.

Other studies have echoed the observations that DBP is associated with bone density in men. Rapado found that DBP concentration was positively associated with lumbar spine BMD in 140 elderly men (aged 55–90) [18]. Non-SNP variants of DBP may also be important in men. One polymorphism is a variable number of (TAAA)_*n*_-Alu sequences in intron 8, which has been associated with an effect on plasma levels of DBP [4]. Al-Oanzi and colleagues studied this polymorphism in 170 male subjects that included 114 healthy males and 56 with idiopathic osteoporosis and low trauma fractures [19]. Specific alleles of the (TAAA)_*n*_-Alu repeat expansion were associated with bone mineral density as well as DBP concentrations. However, studies of DBP and bone health in men have not universally been positive. A study of 211 men over 70 years of age found no association of either the conventional DBP phenotypes or (TAAA)_*n*_ repeat expansion genotypes in BMD or markers of bone turnover [20]. Replication of these genetic associations is key for validating early findings.

## 4. DBP and Vitamin D Bioavailability

The majority of both 25(OH) D and 1,25(OH)_2_ D circulate in large part bound to DBP (85–90%). A lesser fraction is bound weakly to albumin, while less than 1% circulates in its free form [21]. Animal studies suggest that DBP serves to protect 25(OH) D from degradation, prolonging its half-life and protecting against vitamin D deficiency [22]. In addition to stabilizing vitamin D concentrations, however, DBP appears to slow the action of vitamin D in the intestine and reduce uptake by the liver. Additionally, other studies have demonstrated that DBP diminishes the action of vitamin D on target tissues such as monocytes and keratinocytes [23, 24]. These findings have supported the application of the free hormone hypothesis to vitamin D. This model postulates that only hormones that are not bound to the carrier proteins are free to enter cells and induce biological actions [25]. Following the identification of binding coefficients of 25(OH) D and 1,25(OH)_2_ D with albumin and DBP [21, 26], relative fractions of these hormones in bound and unbound states could be calculated from their total concentrations and the concentrations of albumin and DBP. Vitamin D behaves similar to other hormones such as testosterone, where binding to a specific binding protein is much stronger than to albumin. Albumin-bound hormone can be considered similar to free hormone as it is available to act on target tissues, while binding-protein bound hormone cannot. Indeed, given this weak binding, fluctuations in albumin level (e.g., in many disease states) would not be expected to markedly affect vitamin D bioactivity. Our group adapted the previously validated formulae for bioavailable testosterone to vitamin D to define bioavailable vitamin D concentrations [27, 28]. In a study of 49 healthy young adults, we found that bioavailable 25(OH) D (not bound to DBP) was highly correlated with bone density, while total 25(OH) D was not [28].

A follow-up study in a cohort of incident hemodialysis patients similarly found that bioavailable 25(OH) D was positively correlated with serum calcium and negatively correlated with PTH levels, while 25(OH) D displayed no such relationship [29]. In a recent publication, we examined the effects of race and DBP allelic variants in a large cohort of black and white Americans, focusing on the commonly studied SNPs rs7041 and rs4588 [9]. Blacks displayed lower 25(OH) D and DBP levels, but higher BMD. Genetic polymorphism explained the vast majority of the variation in DBP levels (79.4%). Though higher PTH levels were associated with lower 25(OH) D levels as expected, blacks had markedly lower 25(OH) D levels compared with whites within each quintile of PTH. This discrepancy appeared to be explained by assessment of bioavailable 25(OH) D, which was similar between the two races and displayed similar associations with PTH when examined by quintile. Some notable race-specific effects, however, were found. The T variant of rs7041 was associated with lower DBP in both races but lower 25(OH) D levels only in blacks, while the A variant at rs4588 was associated with higher DBP in both races but lower 25D only in whites. BMD was associated with total and bioavailable 25(OH) D levels only in whites. The DBP diplotype associated with the lowest DBP levels (Gc1F/1F) was common in blacks, but rare in whites. These marked racial differences are likely to be key for interpreting and assessing generalizability of studies involving both vitamin D and DBP.

Ongoing controversies remain with the interpretation of DBP levels. Assays are not standardized and can yield different results from one another, either due to differential binding to different DBP isoforms or due to assay nonspecificity [30–32]. While most studies of free or bioavailable vitamin D have relied on calculated values based on binding coefficients, more precise methods for assessing DBP binding are being developed. One recent study included a novel immunoassay that aims to directly measure free 25(OH) D and compared this with the conventional calculated approach in 155 individuals, including some cirrhotic and some pregnant subjects [33]. This study found that, as expected, DBP and albumin levels were the lowest in subjects with cirrhosis but that measured free 25(OH) D was the highest. Despite the higher DBP levels in pregnancy, however, measured free 25(OH) D was not different in pregnant women than in nonpregnant subjects. While calculated and measured free 25(OH) D levels were correlated, this correlation was relatively weak, explaining only 13% of the variability, and was particularly weak in African Americans. The authors also found that only measured and total 25(OH) D levels correlated with PTH. Additional assays to directly assess free and bioavailable vitamin D are under development, and all will require additional validation and comparison with clinical outcomes to determine the optimal assay.

## 5. Summary and Future Directions

A renewed interest in DBP has accompanied a general upswing in vitamin D research in recent years and, indeed, DBP may be key for understanding and interpreting vitamin D's action, particularly with respect to bone health. Numerous studies have found that DBP levels and phenotypic variants correlate with both total 25(OH) D levels and BMD and other measures of bone health. However, these associations have not been consistent in the literature, and the magnitude of these effects is still of unclear clinical significance. Furthermore, many studies of DBP variants have been performed in specific, homogenous populations and how well these findings translate more broadly is not well known at this time. Additional large population studies are needed to better define the role of DBP and its variants on biological outcomes. The concept of bioavailable vitamin D, the fraction of vitamin D not bound to DBP, may explain some of DBPs effects on bone health and will be explored further as dedicated assays are developed. Additional actions, including DBP's actions on monocyte and osteoclast activation, may also be important in determining bone density. As yet unrealized is the potential for drugs that influence DBP or its binding that might be useful in novel approaches to the treatment or prevention of bone disease. This remains in exciting avenue for future investigation.

## Figures and Tables

**Table 1:** Common phenotypic variants of DBP and associated characteristics.

Phenotype	rs7041 (D432E)	rs4588 (T436K)	DBP levels in homozygotes	25(OH) D affinity
GC1F	t (D: asp)	c (T: thr)	Lowest	Highest
GC1S	g (E: glu)	c (T: thr)	Highest	Intermediate
GC2	t (D: asp)	a (K: lys)	Intermediate	Lowest

The three most widely studied variants of DBP include GC1F, GC1S, and GC2, which are distinguished by their SNPs rs7041 and rs4588. The associated nucleotide and amino acid changes are presented, along with known data on DBP levels in homozygotes and affinity for 25(OH) D (derived from Powe et al. [9] and Arnaud and Constans [7]). Conflicting data regarding these relationships remain [10].
